# Factors Responsible for Non-Diagnostic Cytology on Ultrasound-Guided Fine-Needle Aspiration of Thyroid Nodules

**DOI:** 10.7759/cureus.14955

**Published:** 2021-05-11

**Authors:** Muhammad Asad Ullah, Junaid Iqbal, Muhammad Saad Ahmed, Jaideep Darira, Irfan Lutfi, Kamran Hamid, Muhammad Ali

**Affiliations:** 1 Diagnostic Radiology, Dr. Ziauddin Hospital, Karachi, PAK; 2 Radiology, Dr. Ziauddin University Hospital, Karachi, PAK; 3 Interventional Radiology, Shaheed Mohtarma Benazir Bhutto Medical College, Dow University of Health Sciences, Karachi, PAK; 4 Radiology, Dr. Ziauddin Hospital, Karachi, PAK

**Keywords:** non-diagnostic cytology, thyroid nodules, fnac, ultrasound guided

## Abstract

Introduction

Fine-needle aspiration (FNA) is a well-recognized procedure for the diagnosis of thyroid nodules, with the advantage of being safe and inexpensive. Fine-needle aspiration cytology (FNAC) is mainly performed for nodules showing suspicious sonographic features that may require thyroidectomy. Even when FNAC is performed under sonographic guidance, the cytological specimen obtained may be inadequate, leading to a non-diagnostic outcome. The aim of this study is to determine the sonographic and technical factors influencing the outcome of FNAC.

Material and methods

This cross-sectional study was conducted prospectively at the radiology department, Dr. Ziauddin Hospital, Karachi, from January 1, 2019, to December 31, 2020. This study was approved by the Ethical Review Committee (ERC) of Ziauddin University. All the patients undergoing ultrasound (US)-guided FNAC of thyroid nodules were included. Patients with a history of previous thyroid surgery, very large thyroid lesions (>5 cm), and those with adjacent soft tissue pathology obscuring the assessment of thyroid nodules were excluded from this study.

Result

Out of 176 nodules studied, 14 were non-diagnostic and 162 were diagnostic. A 22G needle was used in most of the patients, i.e. 102 (57.3%), which demonstrated no relationship with the non-diagnostic results. According to Bethesda, 136 (77.3%) patients were benign, 22 (12.5%) had lesions with atypia/follicular lesions of undetermined significance, 14 (8%) were non-diagnostic and four (2.3%) were suspicious for malignancy. A subset, including 76 nodules, was categorized according to Thyroid Imaging Reporting and Data System (TIRADS) as follows: 28 (36.8%) nodules were moderately suspicious, 24 (31.6%) were mildly suspicious, 20 (26.3%) were not suspicious, and four (5.3%) nodules were benign. It was also observed that none of the hypoechoic nodules yielded non-diagnostic cytology.

Conclusion

This study concludes that radiologists must be aware of the technical details, cytologic preparation, and procedure-related complications associated with US-guided FNA to optimize patient care and the diagnostic outcome.

## Introduction

A thyroid nodule is a distinct lesion in the parenchyma of the thyroid gland; most are benign and around 5% of excised nodules are malignant [1]. Palpable thyroid nodules are more commonly found in the female gender, with a greater prevalence in the iodine-deficient regions [2]. With ultrasound (US) imaging, the number of detectable thyroid nodules is higher, i.e., nearly 76% [3]. It is necessary to distinguish between benign and malignant nodules for their management [4]. Globally, the frequency of thyroid malignancy is reported to be 16% on histopathology although it is slightly lower in Pakistan (14.3%) [5-6].

According to the Society of Radiologists in Ultrasound (SRU) recommendations, redundant investigations and surgeries should be avoided in patients with benign nodules due to procedure-related complications [7-8]. Fine-needle aspiration (FNA) has diagnostic sensitivity and specificity in the region of 60% and 80%, respectively, with the advantage of being safe and inexpensive [8-9]. Fine-needle aspiration cytology (FNAC) is mainly performed for nodules showing suspicious sonographic features that may require thyroidectomy [10]. FNAC is usually performed only when the nodules are ≥10 mm in size and with microcalcifications; ≥15 mm and solid or with coarse calcifications; ≥20 mm and mixed solid and cystic or for nodules that significantly increase in size since the previous ultrasound [9].

Even when FNAC is performed under sonographic guidance, the cytological specimen obtained may be inadequate, leading to a non-diagnostic outcome [8]. Although the literature suggests that the risk of malignancy with non-diagnostic FNAC is not high, patients with nodules appearing solid in composition on sonographic examination must be considered for surgical excision [9].

It is important to minimize the non-diagnostic rate through the evaluation of associated determinants. The purpose of this study is to assess the sonographic and technical factors influencing the outcome of FNAC.

## Materials and methods

This cross-sectional was conducted in the radiology department, Ziauddin Hospital, Karachi from January 1, 2019, to December 31, 2020. The approval of this study was obtained from the ethical review committee (ERC) of Ziauddin Hospital. All patients with thyroid nodules and between 18 and 75 years of age undergoing ultrasound-guided FNAC were included in this study following informed written consent. Data were acquired by means of a questionnaire using the non-probability consecutive sampling technique. Patients with known history of thyroid surgery, very large thyroid lesions (>5 cm), and having an adjacent soft tissue pathology obscuring the assessment of thyroid nodules were excluded.

FNAC of thyroid nodules was conducted in the procedure room under ultrasound guidance and examined by a radiologist [11]. LP needles of different gauge (ranging from 18 to 25G) attached to a 10-ml syringe holder were used to perform cytological aspirations. A minimum of two needle passes was done for each case. The specimens were expelled onto two or three slides, and thin smears were prepared between two slides and immediately fixed. Sonographic nodular features were evaluated using a 4 to 16 MHz linear array probe of the Toshiba Xario 100 ultrasound machine. This included nodular size, number, shape, margin, echogenicity, echogenic foci, and nodule elements (solid, cystic/complex). Thyroid Imaging Reporting and Data System (TIRADS) developed by The American College of Radiology was used for stratification of thyroid nodules into the following categories: benign (TR1), not suspicious (TR2), mildly suspicious (TR3), moderately suspicious (TR4), and highly suspicious (TR5).

The Bethesda System for Reporting Thyroid Cytopathology (BSRTC) is a standard tool for classifying nodules according to the probability of malignancy. It includes six categories as follows: I - nondiagnostic, II - benign, III - atypia of undetermined significance/follicular lesion of undetermined significance (FLUS), IV - follicular neoplasm (FN)/suspicious for follicular neoplasm (SFN), V - suspicious for malignancy (SM), and VI - malignant. The cytological outcome for each nodule was recorded.

All statistical analyses were performed by using the Statistical Package for Social Sciences (SPSS) version 20 (IBM Corp., Armonk, NY). Quantitative data were expressed as mean $\pm$ SD, whereas qualitative data were expressed as frequency and percentage. The student t-test and chi-square test were applied for outcome variables.

## Results

Among 178 patients included in this study, two nodules were lost to follow-up due to inaccessible cytology results. Out of 176 nodules, 30 were from male patients (17.0%) and 146 from females (83.0%). The mean age of patients was calculated as 44.5 ± 14.0 years. 

Out of 176 nodules studied, 14 were non-diagnostic and 162 were diagnostic. All of the non-diagnostic nodules were identified in female patients, with each nodule from male patients returning a diagnostic cytological outcome (p-value 0.006). A 22G needle was used for FNAC in most of the patients, i.e. 102 (57.3%), which demonstrated no relationship with the non-diagnostic results. According to Bethesda, 136 (77.3%) patients were benign, 22 (12.5%) had lesions with atypia/follicular lesions of undetermined significance, 14 (8%) were non-diagnostic, and four (2.3%) were suspicious for malignancy. None of the nodules turned out to be either suspicious for follicular neoplasm (IV) or malignant (VI) as per Bethesda.

A subset of randomly selected 76 patients with thyroid nodules was evaluated for sonographic features and stratified according to TIRADS. Out of these 76 nodules studied, 28 (36.8%) nodules were moderately suspicious, 24 (31.6%) were mildly suspicious, 20 (26.3%) were not suspicious, and four (5.3%) nodules were benign.

The majority of the nodules had mixed cystic and solid composition (56.5%). There was an indifferent echogenic representation of nodules, with 43.4% hyper to isoechoic nodules and 46.0% hypoechoic nodules. Most of the nodules had smooth margins (62, 86.8%) and none of them had a taller-than-wide configuration.

The association of clinical parameters, TIRADS, and sonographic variables with diagnostic and non-diagnostic FNAC is depicted in Table 1. It was observed that none of the hypoechoic nodules yielded non-diagnostic cytology.

**Table 1:** Association of clinical parameters, TIRADS, and sonographic variables with the diagnostic and non-diagnostic outcomes of FNA Data presented as mean±SD or n (%); ANOVA and the chi-squared test were applied; P-value<0.05 considered to be statistically significant TIRADS: Thyroid Imaging Reporting and Data System; ANOVA: Analysis of Variance; FNA: Fine-Needle Aspiration

Parameters	Non-diagnostic	Diagnostic	P-value	Overall
n	14	162	-	176
Age(years)	44±5.5	44±14.5	0.981	44.5±14
15-30	0(0.0)	26(16.04)	0.135	26(14.77)
31-45	8(57.14)	68(41.97)		76(43.18)
46-60	6(42.85)	47(29.01)		53(30.11)
61-75	0(0.0)	21(12.96)		21(11.93)
Gender				
Male	0(0.0)	30(18.51)	0.077	30(17.04)
Female	14(100))	132(81.48))		146(82.95)
Needle size				
16	2(14.28)	2(1.23)	0.002	4(2.27)
18	2(14.28)	6(3.70)		8(4.54)
20	0(0.0)	4(2.46)		4(2.27)
21	0(0.0)	12(7.40)		12(6.81)
22	6(42.85)	96(59.25)		102(57.95)
23	0(0.0)	26(16.04)		26(14.77)
24	0(0.0)	2(1.23)		2(1.136)
25	4(28.57)	14(8.64)		18(10.22)
TIRADS	Non-diagnostic	Diagnostic	P-value	Overall
n	6	70	-	76
Benign	0(0.0)	4(5.71)	0.092	4(5.26)
Not suspicious	4(66.6)	16(22.85)		20(26.31)
Mildly suspicious	0(0.0)	24(34.28)		24(34.57)
Moderately suspicious	2(33.3)	26(37.1)		28(36.84)
Composition			-	
Cystic	0(0.0)	8(11.42)		8(10.52)
Mixed cystic and solid	4(66.6)	39(55.7)	0.669	43(56.57)
Solid	2(33.3)	23(32.85)		25(34.42)
Echogenicity				
Anechoic	2(33.3)	6(8.57)	0.030	8(10.52)
Hyperechoic or isoechoic	4(66.6)	29(41.42)		33(43.42)
Hypoechoic	0(0.0)	35(50)		35(46.05)
Shape				
Wider-than-tall	6(100)	70(100)	N/A	76(100%)
Margin				
Smooth	4(66.6)	62(85.71)	0.158	66(37.5)
Ill-defined	0(0.0)	2(2.85)		2(2.63)
Lobulated or irregular	2(33.3)	6(8.57)		8(10.52)
Echogenic foci				
None or large comet-tail artifacts	6(100)	56(80)	0.225	62(81.57)
Macrocalcifications	0(0.0)	14(20)		14(18.42)

Table 2 provides an analysis of the association of Bethesda with demographic variables such as age and gender. Though no significant results were obtained, nodules suspicious for malignancy were seen in a slightly older age group (57.5 years ± 17.68).

**Table 2:** Association of age and gender with Bethesda outcome Data presented as n (%); P<0.05 statistically significant

	Bethesda Classification				
Demographics	Non-diagnostic	Benign	Atypia/follicular lesion of undetermined significance	Suspicious for malignancy	P-value
Age(years)	44 (±5.8)	43.63 (±14.22)	48.67 (±13.89)	57.5 (±17.68)	0.417
Male	0(0.0)	20(14.7)	8(36.4)	2(50)	0.006
Female	14(100)	116(85.3)	14(63.6)	2(50)	

Table 3 provides a comparison of TIRADS and Bethesda (p>0.05). No statistically significant association was observed between them.

**Table 3:** Comparison of TIRADS with Bethesda Data presented as n (%); P<0.05 statistically significant; TIRADS: Thyroid Imaging Reporting and Data System

TIRADS	Non-diagnostic	Benign	Atypia/follicular lesion of undetermined significance	Suspicious for malignancy	P-value
Benign (TR1)	0 (0)	4 (6.66)	0 (0)	0(0)	0.142
Not suspicious (TR2)	4(66.66)	12 (20)	4(50)	0(0)	
Mildly suspicious (TR3)	0(0)	22 (36.66)	2(25)	0(0)	
Moderately suspicious (TR4)	2(33.33)	22 (36.66)	2 (25)	2(100)	

## Discussion

Thyroid nodules are frequently found in the Pakistani population. Cytological diagnosis of these nodules may be affected by various factors, including both sonological and technical parameters. Our study demonstrated only 7.95% non-diagnostic FNAC results. This low non-diagnostic percentage corresponds with the proposed focus of under 10% as per the Bethesda System for Reporting Thyroid Cytopathology (BSRTC), highlighting the standardized procedural protocols maintained at our radiology department.

Needle gauge size has a great influence on yielding the cytology of neck and head lesions, including thyroid, salivary glands, and cervical lymph nodes, where larger needle gauges are more likely to reveal hemorrhagic non-diagnostic aspirates [12]. In the present study, although no statistically significant relationship between needle size and the diagnostic outcome of FNA was established, the predominant usage of needles of 22 gauge demonstrated a very low number of non-diagnostic results (six) indicating the positive impact of smaller gauge size on diagnostic cytology rates [13].

Even though ultrasound guidance facilitates the secured targeting of thyroid nodules, the rate of non-diagnostic FNA is estimated as between 0.4% and 40.7% [14-15]. According to the literature, 2.0%-14% of non-diagnostic nodules proved to be malignant on surgically excised tissues [16-17]. Therefore, non-diagnostic fine needle aspiration outcomes should not be regarded as simply benign. To avoid delayed recognition of malignant thyroid nodules and unnecessary surgeries, the professional medical societies recommended a repeat fine-needle aspiration under ultrasound guidance for all non-diagnostic nodules [18-19]. Instead, a core needle biopsy is endorsed as an alternative procedure to improve the diagnostic outcome of ultrasound-guided FNA [20].

Factors that contribute towards the non-diagnostic outcome of US-guided FNA depend on the nodular features and operator’s experience besides cytologic preparation and interpretation of FNA specimen [13]. Cystic dominancy, macro-calcification, size less than 5-10 mm, or hypo-echogenicity more often yielded non-diagnostic results according to previous studies [21]. We studied the association of nodule echogenicity with non-diagnostic and diagnostic FNA outcomes, concluding that though very limited, non-diagnostic FNAC results were not observed among hypoechoic nodules. This is contrary to the current literature and needs further evaluation in future studies in larger groups. Most of the US-guided FNA performed by experienced operators revealed cystic dominance and intra-nodular macro-calcification as independent factors for non-diagnostic results [21].

Additionally, published studies depicted that 2.2%-2.5% of thyroid cancers had prominent cystic changes alongside at least one suspicious feature, for example, micro-calcification, eccentric solid nodule, hypo-echogenicity, or irregular thick wall [22]. Therefore, the FNA specimen must target correctly the internal solid portion following drainage of cystic content.

In our study, TIRADS classification did not exhibit a congruent relationship with the cytological diagnosis of thyroid nodules. A previous study reported an association of TIRAD class V with malignant thyroid nodules while TIRADS II, III, and IVa with benign thyroid nodules on FNAC [23]. In the current study, results show a decline in non-diagnostic rates for FNAC executed under radiological assistance when compared to non-diagnostic rates reported in recent literature. Further large-scale studies involving multiple centers and consideration of other confounding factors is needed to validate the findings of this study.

## Conclusions

This study concludes that smaller needle sizes decrease the likelihood of the non-diagnostic outcome of FNAC. Our study further showed that the cytology of none of the hypoechoic nodules culminated as non-diagnostic. We propose that the multidisciplinary team involved in the management of thyroid nodules, including radiologists, must be aware of the technical details, cytologic preparation, and standardized procedural protocols for US-guided FNA to optimize the diagnostic outcome of US-guided FNA and augment patient care.
